# Glucosinolate Profiles in Cabbage Genotypes Influence the Preferential Feeding of Diamondback Moth (*Plutella xylostella*)

**DOI:** 10.3389/fpls.2017.01244

**Published:** 2017-07-18

**Authors:** Arif Hasan Khan Robin, Mohammad Rashed Hossain, Jong-In Park, Hye R. Kim, Ill-Sup Nou

**Affiliations:** ^1^Department of Horticulture, Sunchon National University Suncheon, South Korea; ^2^Department of Genetics and Plant Breeding, Bangladesh Agricultural University Mymensingh, Bangladesh; ^3^Plant Systems Engineering Research Center, Korea Research Institute of Bioscience and Biotechnology Daejeon, South Korea

**Keywords:** cabbage, glucosinolates, resistance, Diamondback moth, insect preference

## Abstract

Diamondback moth (DBM), *Plutella xylostella* L., is a devastating pest of cabbage worldwide whose feeding attributes are influenced by glucosinolate profiles of the plant. Identifying the specific glucosinolates associated with plants’ resistance mechanism can provide cues to novel points of intervention in developing resistant cultivars. We studied the DBM larval feeding preference and extent of damage on cabbage leaves via controlled glass-house and *in vitro* multiple- and two-choice feeding tests. These feeding attributes were associated with the individual glucosinolate profiles, analyzed by HPLC, of each of the eight cabbage genotypes using multivariate analytical approach to identify the glucosinolates that may have roles in resistance. Both the glass-house and *in vitro* multiple-choice feeding tests identified the genotype BN4303, BN4059, and BN4072 as the least preferred (resistant) and Rubra, YR Gold and BN3383 as most preferred (susceptible) genotypes by DBM larvae. The principal component analysis separated the genotypes based on lower feeding scores in association with higher contents of glucobrassicin, glucoiberin, glucoiberverin in one direction and 4-hydroxyglucobrassicin, glucoerucin, glucoraphanin, and progoitrin in opposite direction in a way to explain the major variation in resistant versus susceptible genotypes based on their extent of preference and leaf area damage. The simultaneous presence (or higher contents) of glucobrassicin, glucoiberin, and glucoiberverin and the absence (or lower contents) of 4-hydroxyglucobrassicin, glucoerucin, glucoraphanin, and progoitrin in the least preferred genotypes and *vice-versa* in most preferred genotypes indicated their apparent role as putative repellents and attractants of DBM larvae in cabbage genotypes, respectively. These novel findings add to the current knowledgebase on the roles of glucosinolates in plant–herbivore interactions and will be helpful in setting breeding priorities for improving the resistance against DBM in cabbage using conventional and biotechnological approaches.

## Introduction

*Brassica oleracea* is one of the most important human food crop species commonly consumed as vegetables, with a range of commercially valuable subspecies that includes cabbage, cone cabbage, cauliflower, broccoli, gai lan, kale, kohlrabi, collard greens, savoy, and Brussels sprouts etc. Cabbage are naturally rich in potassium, fiber, folic acid, beta-carotene, vitamins C, and K while containing less fat, cholesterol, and sodium. This make it important in terms of health, medical, and clinical benefits as evident by their protective role against cancer, heart disease, diabetes, and hypertension (van Poppel et al., 1999; Finley, 2003; King and Barker, 2008; Verkerk et al., 2009; Kapusta-Duch et al., 2012). *Brassica* production, however, is affected by a number of abiotic and biotic stresses including the oligophagous Diamondback moth (DBM) *Plutella xylostella* L. (Plutellidae, Lepidoptera), a highly devastating pest that specializes on *Brassica* (Henniges-Janssen et al., 2014; Moreira et al., 2016). DBM causes an estimated global loss of US$ 1 billion in direct losses and control costs (Sarfraz et al., 2006; Furlong et al., 2013). Besides, chemical control of the pest were proven cost ineffective and environmentally hazardous along with the chances of rapid development of resistance toward insecticides and potential negative effects on their natural enemies (Grzywacz et al., 2010). Development of resistant varieties can thus be the most sustainable approach which requires wider understanding of the plants overall defense mechanisms with regards to the natural variation in plants’ performances against DBM.

Synthesis of a plethora of bio-chemical compounds that act directly on insect herbivores or via attracting their natural predators and parasitoids, is a common defense strategy of plants (Fatouros et al., 2005; Scascighini et al., 2005; Ode, 2006; Kos et al., 2011; Mithöfer and Boland, 2012). Brassicaceae plants particularly use glucosinolates-myrosinase defense system against insects and pathogens (Talekar and Shelton, 1993; Bartlet et al., 1999; Henniges-Janssen et al., 2014; Santolamazza-Carbone et al., 2014). Glucosinolates, the sulfur-rich secondary metabolites, widely synthesized in all vegetable and oilseed species of the order Brassicales including *Brassica oleracea*, represent classical examples of plant compounds known for their role in insect–plant interactions (Mithen et al., 1995; Benderoth et al., 2006; Hopkins et al., 2009).

More than 120 different glucosinolates are known that can be grouped into one of three major structural classes: indolics (constitute about 10% of glucosinolates and are synthesized from tryptophan), aliphatics (constitutes 50% of glucosinolates and are synthesized from methionine, alanine, valine, leucine, and isoleucine), and aromatics (constitutes 10% of glucosinolates and synthesized from phenylalanine or tyrosine) (Fahey et al., 2001; Mithen, 2001; Bekaert et al., 2012). The enzyme myrosinase, a thioglucosidase usually stored in special myrosinase cells in all plant organs comes into contact with glucosinolates when the tissue damage is commenced due to insect feeding. This enzyme hydrolyses indole glucosinolates to produce nitriles and unstable isothiocyanates and aliphatic glucosinolates to produce volatile and pungent isothiocyanates. These hydrolysis products of glucosinolate compounds have toxic properties that inhibits growth (antibiosis) and act as feeding deterrents (antixenosis) against a range of insects; from leaf chewing lepidopteran larvae to phloem-feeding aphids (Santolamazza-Carbone et al., 2016). These also negatively influence the development and fitness of generalist insects by exerting decreased food efficiency, poor palatability and growth depression etc. (Hopkins et al., 2009). Thus, the hydrolysis products of glucosinolates enhance the defensive properties in conferring resistance against insect herbivores (Rask et al., 2000; Mithen, 2001; Bones and Rossiter, 2006; Burow et al., 2006).

Constitutive levels of glucosinolates act as important defense molecules as evident by their direct association with damage levels upon insect feeding (Bidart-Bouzat and Kliebenstein, 2008). However, the role of glucosinolate in plant–herbivore interaction remain dynamic due to their differential influence on generalists and specialists insects (Lambdon and Hassall, 2005; Bidart-Bouzat and Kliebenstein, 2008; Hopkins et al., 2009; Santolamazza-Carbone et al., 2014, 2016). Glucosinolates and their breakdown products are generally considered to be potential feeding deterrent for generalist insect species, and contrastingly, can typically stimulate host plant acceptance by acting as oviposition and feeding stimulants (kairomonal role) for *Brassica* specialists of the genera Coleoptera, Lepidoptera, and Diptera (Rosenthal and Berenbaum, 1991; Mithen et al., 1995; Wittstock et al., 2003; Schoonhoven et al., 2005; Renwick et al., 2006). More to this, the specialist insect DBM is reported to feed on both low and high glucosinolate containing lines of *B. juncea* (Bodnaryk, 1997). Ratzka et al. (2002) reported the ability of DBM larvae to adapt specifically to brassicaceous plants by overcoming their chemical defenses using a highly active glucosinolate-sulfatase. Additionally, the moths even use some glucosinolates and their breakdown products as host identification and oviposition stimulants (Ratzka et al., 2002; Sarfraz et al., 2006; Müller et al., 2010). Contrastingly, the relative growth rates of the generalist *Spodoptera eridania* and the specialist *P. xylostella* were reduced on *Brassica juncea* homozygous lines with high glucosinolate concentrations (Li et al., 2000). Responses of herbivores to glucosinolates further depend on the glucosinolate class. High sinigrin content decreased the abundance of both specialist DBM and generalist *Mamestra brassicae* in *Brassica oleracea* var. *acephala* (Santolamazza-Carbone et al., 2016). High levels of indole glucosinolate, neoglucobrassicin slowed the development in *Pieris rapae* whereas the high levels of the aliphatic glucosinolates gluconapin and sinigrin reduced the survival of *Mamestra brassicae*.

The patterns of insect herbivory were thus found to be intrinsically influenced by both quantitative and qualitative variation in various glucosinolate profiles in the Brassicaceae making the issue particularly complicated (Lambrix et al., 2001; Windsor et al., 2005; Cartea et al., 2008; Poelman et al., 2008, 2009; Lankau and Kliebenstein, 2009; Kos et al., 2011). The natural variation in glucosinolate profiles in *Brassica* and the complex interaction of various insect herbivores requires in depth species- and insect-specific characterization of individual glucosinolates. Direct evidence of such in depth studies to identify the role of individual glucosinolate compounds in deterring DBM remains scarce. Investigating the association between individual glucosinolate compounds and preferential feeding behavior of DBM will thus be helpful in identifying the key glucosinolate determinants of resistance against DBM which will serve as important biomarker for setting breeding priorities for developing DBM resistant cabbage varieties.

## Materials and Methods

### Cabbage Genotypes and Plant Growth Conditions

Eight genotypes of *Brassica oleracea* var. *capitata* (cabbage) consisting three commercial cultivars namely, Rubra, YR gold, and Ohgane and five inbred lines namely, BN3383, BN4059, BN4072, BN4098, and BN4303 were obtained from Asia Seed Co., Ltd., Seoul, South Korea (Supplementary Figure S1). The genotypes were chosen based on their diverse pedigree records and contrasting morphological properties and prior reputation of differential glucosinolate profiles (Robin et al., 2016). Seeds were germinated and seedlings were raised in moistened garden soil mix at 25 ± 1°C, 65% relative humidity, 80–120 μmol m^-2^s^-1^ light intensity and 16 h photoperiod in a controlled plant growth room. After 4 weeks when seedlings attained a height to withstand transplantation shock, seedlings were transferred to 30 cm × 25 cm × 15 cm garden pots filled with a mixture of 1:3 sand/commercial potting mix 25 cm apart in glasshouse and maintained by light irrigation, standardized NPK fertilization, and without using pesticides before collecting leaf samples for glucosinlate profiling, *in vitro* feeding tests and insect imposition for glass-house feeding test at 3 months of total age.

### Collection and Rearing of Diamondback Moth

Diamondback moth, *Plutella xylostella* L., were collected from nearby natural field using sweep nets and poolers and were harbored on *Brassica napus* plants inside a 2.5 m^2^ makeshift net cage in glass-house (20–25°C temperature, 60–70% RH, ambient light level, natural photoperiod) prior to being used to assess their preferential feeding behavior on cabbage genotypes. For *in vitro* feeding assessment, the eggs were allowed to hatch and the neonate larvae were reared in plastic boxes in controlled growth room and fed with fresh *B. napus* leaves until they reached fourth instar stage.

### Insect Herbivory in Glass-House Experiment

**FIGURE 1:**
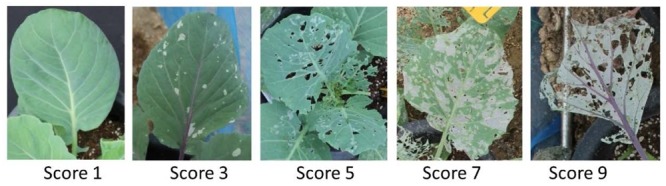
Visual representation of feeding scores used to assess the extent of leaf damage in net cage of glass-house by DBM larvae. The score ranged from ‘1’ to ‘9’ with ‘1’ being scored for leaves with no damage symptoms and ‘9’ being scored for maximum damaged leaf.

Three months old cabbage plants grown in the glass-house were transferred to four net cages (tents) where adult DBM were being reared on *B. napus* plants. The insect population inside the net was approximately 50 adult m^-2^. One plant from each of the eight varieties were planted 25 cm apart and randomly in a net cage. Four net cages thus represent four replications set with randomized complete block design (tent experiment). Insect herbivory in each cabbage genotypes were measured in terms of percent leaf area damaged by DBM larvae at 2, 4, 7, and 13 days after insect introduction using a visual score of 1–9 where ‘1’ and ‘9’ being scored for least and maximum area damaged as shown in **Figure 1**. The higher scores thus indicate a higher feeding preference of adult Diamondback moth on the cabbage genotypes. The percent leaf area damaged by the DBM larvae was measured based on visual estimation using eight replications per genotypes.

### *In Vitro* Insect Herbivory

**FIGURE 2:**
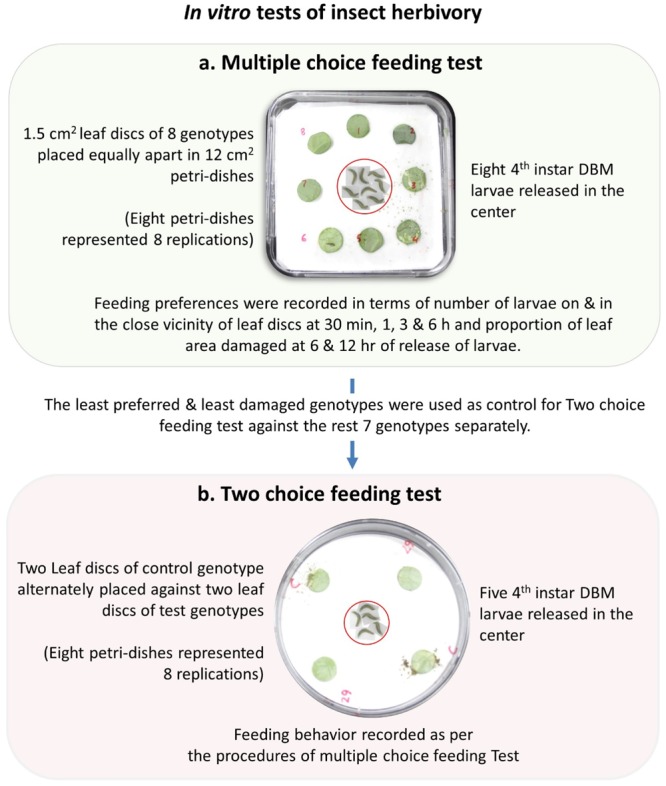
Detailed procedure of *in vitro* insect herbivory used in the multiple-choice and two-choice feeding tests of fourth instar Diamondback larvae on the cabbage genotypes.

Infestation and damage by insects were also tested *in vitro* using two different procedures namely, multiple-choice feeding test and two-choice feeding test. For multiple-choice feeding test, leaf disks of 1.5 cm diameter were cut out from each genotypes and placed equally apart in square plastic petri-dishes (12 cm × 12 cm × 2 cm). Eight fourth instar larvae were released in the center of the petri-dish and their feeding preferences were measured in terms of number of larvae on and in the close vicinity of the leaf disk of each of the eight genotypes at 30 min, 1, 3, and 6 h and percentage of leaf area damaged at 6 and 12 h of release of larvae (**Figure 2**). For two-choice feeding test, insect herbivory of each genotype was tested separately against the control genotype, BN4303. Two leaf disks of each test genotype were placed alternately against two leaf disks of control genotype in one plastic petri-dish. Four larvae were released in the center of each petri-dish and their preferential feeding behavior was assessed based on similar parameters of multiple-choice *in vitro* procedures as described above. Data of both multiple- and two-choice feeding tests were recorded from eight replications. The genotype BN4303 was chosen as control genotype due to its least preference (least damage occurred) to DBM as per glass-house and *in vitro* multiple-choice feeding test.

### Glucosinolate Profiling Using HPLC

For profiling constitutive individual glucosinolate compounds in the leaves of eight cabbage genotypes, a slightly adapted version of the HPLC procedures used by Robin et al. (2016) and Yi et al. (2016) were used in this study, which in brief is described here. Methanol treated and frozen leaf tissues (10 g) were grinded to a fine powder and incubated initially at 70°C for 10 min and then at room temperature for 1 h. The structural components and proteins of the tissues were removed by centrifuging the samples at 14000 rpm for 10 min at 4°C. The supernatant was used to conduct an anion-exchange chromatography. This centrifugation and chromatography were repeated three times and the supernatants were pooled which represented the crude extract of glucosinolates. This crude glucosinolates were then desulfated by mixing 0.5 mL 50 mM barium acetate and 0.5 mL 50 mM lead acetate; centrifuging at 2,000 × *g* for 10 min; loading into a 0.5 M sodium acetate pre-equilibrated DEAE-Sephadex column and adding 250 μL aryl sulfatase to the column. After running the desulfation process for 16 h the desulfated glucosinolates were eluted with 1 mL of distilled water which was further purified by centrifugation at 20,000 × *g* for 4 min at 4°C and filtration through a PTFE filter (13 mm, 0.2 μm, Advantec, Pleasanton, CA, United States). The HPLC analysis of the purified desulfo-glucosinolate samples was conducted in a Waters 2695 HPLC system (Waters, Milford, MA, United States) with a C18 column (Zorbax Eclipse XBD C18, 4.6 mm × 150 mm, Agilent Technologies, Palo Alto, CA, United States) using acetonitrile and water as mobile phase solvents. The purified desulfo-glucosinolates were detected using a PDA 996UV-visible detector at a wavelength of 229 nm and quantified using the standard curve of sinigrin. The glucosinolate profiling was concluded by identifying individual glucosinolate molecules (Supplementary Table S4) using Mass spectrometry analysis (HPLC/MS, Agilent 1200 series, Agilent Technologies).

### Statistical Procedures and Principal Component Analysis

Depending on the type and corresponding distribution of the data, combinations of significance tests and *post hoc* tests were performed. Normality of data was tested following Anderson-Darling test in MINITAB v. 17 statistical packages (Minitab Inc., State College, PA, United States). Glucosinolate contents were analyzed using one-way ANOVA and Tukey’s pairwise comparisons in MINITAB v. 17 statistical packages. Glass-house feedings scores were analyzed using Non-parametric Mood’s Median test and *post hoc* Bonferroni’s pairwise comparisons were done for each of the time-points independently using Minitab software packages (v. 17.0). The number of larvae per leaf disk was analyzed using generalized linear model with Poisson distribution using SPSS software packages (v. 24.0). Both genotypic difference and time-point difference were considered as fixed effect. Percent leaf area damage was analyzed using two-way ANOVA and Tukey’s pairwise comparisons using Minitab software packages (v. 17.0). The traits such as glucosinolate content, number of larvae per leaf disk and percent leaf damage were presented as mean ± SD whereas glass-house feeding scores were presented as median and range. The detailed and trait-wise procedure of the statistical analyses are given in Supplementary Table S1. Normality and deviance of data, degrees of freedom, test statistic, and *p*-value of statistical significance of different traits are given in Supplementary Table S2.

Principal component analysis (PCA) was performed using the standardized (mean subtracted from the variable and then divided by the standard deviation) data of individual glucosinolate contents and the glass-house feeding scores at day 7 and 13 and both multiple- and two-choice *in vitro* feeding scores (of 12 h) as set of variables using MINITAB v. 17 statistical packages (Minitab Inc., State College, PA, United States).

## Results

### Genotypic Variations in Glucosinolate Profiles

**FIGURE 3:**
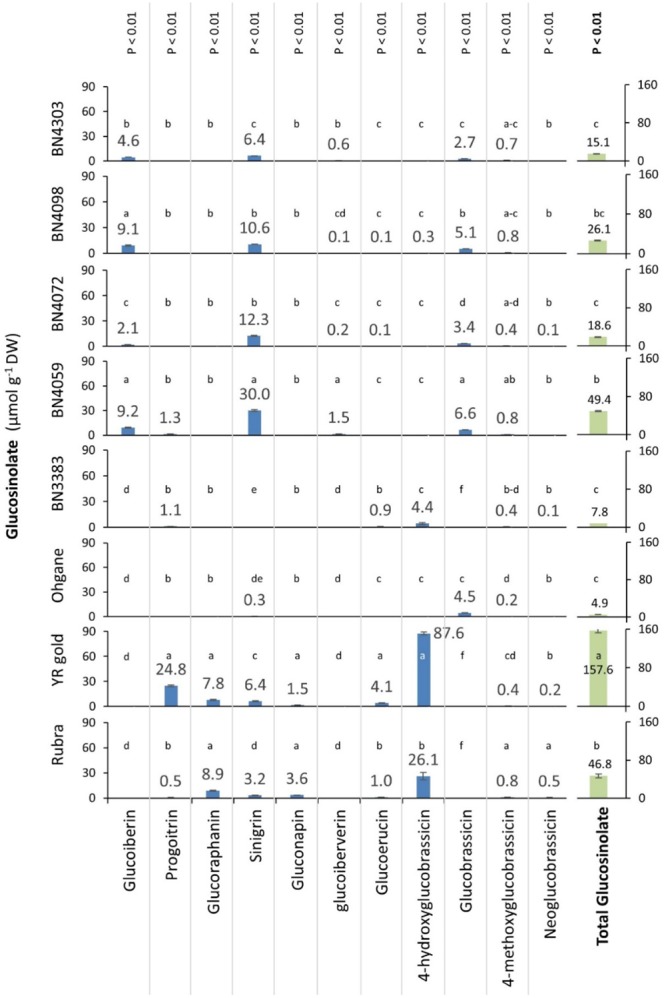
Constitutive glucosinolate contents (μmol g^-1^ dry weight) in the leaf samples of eight cabbage genotypes. Leaf tissue was harvested from the middle aged fully grown leaves of the 3 months old cabbage before insect infestation and analyzed using HPLC (Waters 2695, Milford, MA, United States) and Mass Spectrophotometry (Agilent 1200 series, Agilent Technologies) systems. Data presented as mean (*n* = 3 ± SE). Genotypes varied significantly for individual and total glucosinolate contents (*p* < 0.01) as determined by one-way ANOVA. Different letters (a, b, c, etc.) across genotypes indicate statistically significant differences for glucosinolate contents as per Tukey’s pairwise comparisons.

HPLC analysis of the leaves of three commercial cultivars namely, Rubra, YR gold, and Ohgane and five inbred lines namely, BN3383, BN4059, BN4072, BN4098, and BN4303 detected in total 11 different glucosinolates of which seven were aliphatic glucosinolates namely, sinigrin, glucoiberin, glucoiberverin, glucoraphanin, gluconapin, glucoerucin, and progoitrin and four were indolic glucosinolates namely, glucobrassicin, neoglucobrassicin, 4-methoxyglucobrassicin and 4-hydroxyglucobrassicin (**Figure 3**). Statistically significant differences were observed between the genotypes for individual and total glucosinolate contents (*p* < 0.01 for all) (**Figure 3**). Among these 11 glucosinolate compounds, 4-methoxyglucobrassicin was found in all eight genotypes; in traces (0.2–0.8 μmol g^-1^ DW) in content (**Figure 3**). The aliphatic glucosinolate sinigrin was found in all genotypes except BN3383 with the highest (30 μmol g^-1^ DW) and the lowest (0.3 μmol g^-1^ DW) were being found in BN4059 and Ohgane, respectively.

Contrastingly, glucoraphanin, and gluconapin were found only in two genotypes Rubra and YR Gold. Except glucoiberin, glucoiberverin, and glucobrassicin, all of the rest of the 11 glucosinolates were detected in the genotypes, Rubra and YR Gold. The genotypes, YR Gold contained the highest (157.6 μmol g^-1^ DW) total glucosinolate followed by BN4059 (49.4 μmol g^-1^ DW) and Rubra (46.8 μmol g^-1^ DW) which was characterized largely by the higher concentration of 4-hydroxyglucobrassicin (87.6 μmol g^-1^ DW) and progoitrin (24.8 μmol g^-1^ DW) in YR Gold, sinigrin in BN4059 (30.0 μmol g^-1^ DW) and 4-hydroxyglucobrassicin in Rubra (26.1 μmol g^-1^ DW). The genotype Ohgane (4.9 μmol g^-1^ DW) followed by BN3383 (7.8 μmol g^-1^ DW) contained the lowest total glucosinolate (**Figure 3**).

### Glass-House Feeding Test: Preferential Feeding Behavior of DBM Larvae

**FIGURE 4:**
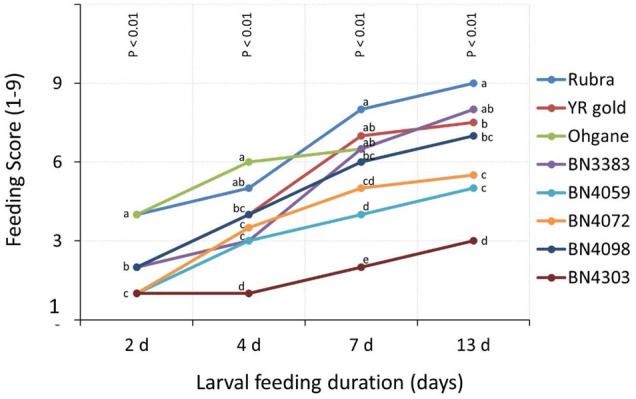
Extent of insect damage on cabbage genotypes measured in terms of visual scores on a scale of 1–9, with ‘1’ being scored for the least and ‘9’ for the highest area damaged by Diamondback moth (DBM) larvae in eight genotypes of cabbage in glass-house experiment. The higher scores indicate a higher feeding preference of adult DBM on the cabbage genotypes. Data presented as median scores of eight observations. The range values are shown in Supplementary Table S2. Genotypes varied significantly for feeding scores (*p* < 0.01) at each time points as determined by Non-parametric Mood’s Median test. Different letters across genotypes at each time points indicate statistically significant differences as per Bonferroni’s pairwise comparisons.

Feeding scores in glass-house experiments increased with the increase in the duration of larval feeding in all genotypes (**Figure 4**). The genotypic differences were significant in each of the time points (*p* < 0.01) for feeding scores as determined by the Non-parametric Mood’s Median test (**Figure 4**). Data revealed that genotypic differences became greater at progressively later time-points compared to earlier time-points. Feeding scores of genotype BN4303 and BN4072 were not significantly different at 2 days after infestation, while they became significantly different at the subsequent time-points (**Figure 4**). At 2 days of larval feeding, the genotypes BN4303, BN4059, and BN4072 were least damaged (**Figure 4** and Supplementary Table S3). Among these three initially least damaged genotypes, BN4303 outstands from the rest of the eight genotypes as the most resistant genotype with its lowest feeding score (3 on a scale of 1–9) making it statistically different form the rest of the genotypes between 4 and 13 days of larval feeding. The other two genotypes, BN4059 and BN4072 also appeared as less damaged with the feeding scores of 5 and 6 (median values), respectively, at 13 days of larval feeding (**Figure 4** and Supplementary Table S3). Notably, all three commercial cultivars, Rubra, YR Gold, and Ohgane appeared as the most damaged by DBM larvae with Rubra and Ohgane showing the consistent signs of maximum damage during the entire larval feeding duration (**Figure 4** and Supplementary Table S3).

### *In Vitro* Feeding Tests: Multiple- and Two-Choice Feeding Behavior of DBM Larvae

**FIGURE 5:**
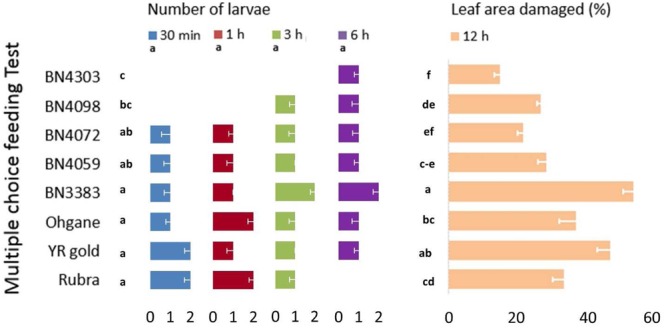
Genotypic variation in eight cabbage genotypes in terms of feeding preference and extent of leaf damage by DBM larvae as determined by *in vitro* multiple-choice feeding test. Data recorded at different time points and presented as mean (*n* = 8). The error bars indicate standard deviations. Different letters across genotypes and time-points indicate statistically significant differences for number of larvae and percent leaf area damaged over all time points and genotypes, respectively, as per Tukey’s pairwise comparisons.

In *in vitro* multiple-choice feeding test, cut leaf disks of all eight genotypes were placed equally apart giving the eight fourth instar DBM larva to preferentially choose from and feed on (**Figure 2**). The percentage of damaged area per leaf disk varied significantly (*p* < 0.01) but the number of larvae per leaf disk did not show any statistically significant variation across different time-points (*p* = 1.00). The genotypic responses, however, varied significantly in terms of both the number of larvae per leaf disk and % leaf area damaged (*p* < 0.01 for both) (**Figure 5**). The most resistant genotype BN4303 strikingly varied from the four most susceptible genotypes Rubra, Ohgane, YR Gold, and BN3383 in terms of both number of larvae per leaf disk and % leaf area damaged (**Figure 5**). The least number of larvae fed on (or visited) BN4303 leaf disks within first 3 h and hence, the area of leaf disks were found to be least damaged (only 5%) within the first 6 h (data not shown) on that genotype (**Figure 5**). BN4098 was the second least visited genotype by DBM larvae within first 3 h causing 26.2% damage to the leaf disk at 12 h of infestation (**Figure 5**). DBM larvae seem to instantly prefer the leaf disks of Rubra and YR Gold as two larvae fed on (or visited) within first half an hour causing a final leaf damage of 33 and 46 percent at 12 h of larval infestation (**Figure 5**). During the entire period of infestation, the maximum number of larvae fed on the leaf disks of BN3383 and hence, inflicted maximum damage (53.3%) at 12 h of infestation (**Figure 5**). Overall, the findings corroborate with that of the glass-house feeding tests as both test identified BN4303, BN4372 as least preferred and BN3383, Rubra, YR Gold, and Ohgane as most preferred genotypes. There were significant variations in the numbers of larvae visited at different time points within genotypes for most of the cabbage lines (*p* < 0.05) except BN4072 and BN4059 (**Figure 5**). The most susceptible genotype Rubra had no larvae visitors at the final studied time point (6 h) whereas it was heavily infested at the initial two time points (*p* < 0.01). In contrast, two resistant genotypes BN4303 and BN4098 had no larvae visitors at the initial time points (30 min and 1 h) whereas a few larvae visited these genotypes at the later time points (*p* < 0.01). This may be because the most susceptible genotypes were heavily damaged initially leaving less leaf area to feed on. This forced the larvae to move on and start feeding the intact leaves of the comparatively less preferred genotypes.

**FIGURE 6:**
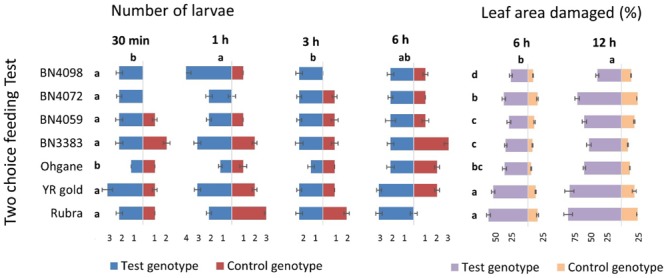
Genotypic variation in seven test cabbage genotypes compared to the control genotype, BN4303 in terms of feeding preference and extent of damage by DBM larvae as determined by *in vitro* two-choice feeding test. Data recorded at different time points and presented as mean (*n* = 8). The error bars indicate standard deviations. Different letters across the seven test genotypes and time-points indicate statistically significant differences (*p* < 0.01) for number of larvae and percent leaf area damaged (letters given in left side of the % leaf area damaged data) as per Tukey’s pairwise comparisons.

In *in vitro* two-choice feeding test, the cut leaf disks of each of the seven genotypes were tested against the leaf disks of control genotype BN4303 (most resistant as per both glass-house and *in vitro* multiple-choice feeding tests) separately to determine the preferential (comparative) feeding behavior of DBM larva (**Figure 2**). Genotypic differences for number of larvae and percent leaf area damaged across different time-points were statistically significant (*p* < 0.01) (**Figure 6**). Number of larvae visitors per leaf disk were significantly higher and percent insect damage was significantly lower in all seven test genotypes compared to control genotype BN4303 (*p* < 0.01). Only the genotype Ohgane differed significantly from the rest of the six test genotypes in terms of mean number of larvae per leaf disk (**Figure 6**). For percent leaf area damaged, the genotypes Rubra and YR Gold were more severely damaged compared to other six test genotypes (**Figure 6**). Like the previous two tests, insects seem to prefer Rubra and YR Gold compared to other genotypes over control genotype BN4303 as evident by higher number of larva visitors that causing almost the complete damage of the leaf disks (82 and 80% area damaged, respectively) during different time points of entire feeding period (*p* < 0.01, **Figure 6**). The highest number of larvae visited YR gold at 30 min and BN4098 at 1 h compared to all other genotypes (*p* < 0.01, **Figure 6**). Genotype BN3383 which appeared as one of the susceptible genotypes in the previous two tests was also found to be visited by more number of larvae at 1 h compared to control, Ohgane, Rubra, BN4059, and BN4072 (*p* < 0.01, **Figure 6**). The use of BN4303 as control genotype was justified as the leaf disk of this genotype was only damaged to a maximum of 25% (range 10–25%) against any of the rest seven genotypes separately, whereas the damage in test genotypes ranged from 37 to 82% during the 12 h of larval feeding (**Figure 6**).

### Association between Genotypic Glucosinolate Profiles and Insect Feeding Behavior

Principal component analysis of the contents of 11 individual glucosinolates and feeding test data extracted major contrast between individual glucosinolates and insect preference toward particular cabbage genotypes. The first three PCs explained 81.5% of the total variation in the datasets (**Table 1**). PC1 accounted for 48.32% of the total variation which is largely manifested by higher positive coefficients for feedings scores or percent leaf damage, 4-hydroxyglucobrassicin, glucoerucin, glucoraphanin, and progoitrin versus lower negative co-efficients for glucobrassicin, glucoiberin, and glucoiberverin (**Table 1**).

**Table 1:** Component loadings of glass house- and *in vitro-* feeding tests; glucosinolate concentrations and mean PC scores of eight cabbage genotypes as determined by the principle component analysis (PCA).

Variable	PC1	PC2	PC3
Feeding score in glass-house (Day 7)	0.278	–0.245	0.065
Feeding score in glass-house (Day 13)	0.239	–0.305	0.014
*In vitro* M-C feeding damage (12 h)	0.175	0.003	–0.304
*In vitro* T-C feeding damage (12 h)	0.235	–0.018	0.237
Glucoiberin	–0.265	0.194	0.221
Progoitrin	0.247	0.372	–0.070
Glucoraphanin	0.286	–0.019	0.328
Sinigrin	–0.172	0.318	0.342
Gluconapin	0.227	–0.201	0.411
Glucoiberverin	–0.233	0.242	0.264
Glucoerucin	0.295	0.274	–0.046
4-Hydroxyglucobrassicin	0.296	0.266	0.027
Glucobrassicin	–0.288	0.145	0.092
4-Methoxyglucobrassicin	–0.087	–0.097	0.494
Neoglucobrassicin	0.177	–0.228	0.250
**Total glucosinolate**	**0.257**	**0.352**	**0.099**
% Variation explained	48.2	20.3	13.0
*P*-value	<0.001	<0.001	<0.001

**Cabbage lines**	**Mean PC scores (±SD)**		

Rubra	1.71 ± 0.51 b	–2.23 ± 0.18 e	1.67 ± 0.42 a
YR gold	3.61 ± 0.60 a	2.29 ± 0.37 a	–0.34 ± 0.65 b
Ohgane	–1.30 ± 0.14 cd	–1.17 ± 0.03 d	–1.87 ± 0.23 c
BN3383	–0.34 ± 0.28 c	–1.35 ± 0.05 d	–2.05 ± 0.63 c
BN4059	–4.66 ± 0.38 g	2.57 ± 0.23 a	2.15 ± 0.16 a
BN4072	–2.33 ± 0.11 de	0.09 ± 0.06 c	–0.25 ± 0.31 b
BN4098	–2.97 ± 0.15 ef	0.05 ± 0.13 c	0.33 ± 0.04 b
BN4303	–4.00 ± 0.04 fg	0.96 ± 0.03 b	–0.25 ± 0.36 b

*M-C, multiple-choice; T-C, two-choice.*

**FIGURE 7:**
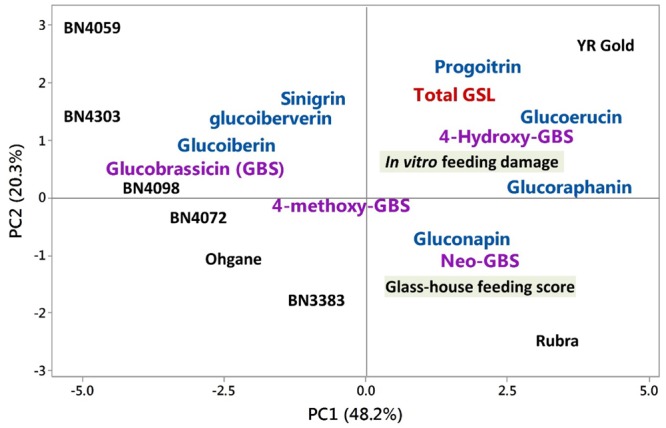
Biplot of glucosinolate contents in eight cabbage genotypes and insect feeding responses as determined by the principle component analysis (PCA). Black letters denote mean PC scores of each cabbage line. Blue and violet colors represent co-efficients between PC1 and PC2 for aliphatic and indolic glucosinolates, respectively. GBS, glucobrassicin; GSL, glucosinolate. Rubra, YR gold, and Ohgane are commercial cabbage cultivars. The other three genotypes with ‘BN’ prefixes are the cabbage inbred lines. *In vitro* and glass-house feeding scores indicate the insects’ preference to cabbage lines.

In PC1, the traits such as the contents of 4-hydroxyglucobrassicin, glucoerucin, glucoraphanin, glucobrassicin, and glass-house feeding score at 7 days; and in PC2, the traits such as the contents of progoitrin, sinigrin and glass-house feeding score at 13 days contributed the most in explaining the overall variability of the entire datasets (**Table 1**). The PC1 clearly separated YR Gold and Rubra (two susceptible genotypes as per feeding tests results) from rest of the genotypes and placed the two most resistant genotypes, BN4303 and BN4059 to the furthest opposite quadrant in PCA-biplot (**Figure 7**). This is also evident from the higher mean PC scores in opposite direction in these resistant (+3.61 and +1.71 in YR Gold and Rubra, respectively) and susceptible (-4.66 and -4.00 in BN4059 and BN4303, respectively) genotypes (**Table 1**).

PC2 explained 20.3% of total variation which is largely dominated by higher coefficients (and also higher contents) of aliphatic progoitrin, sinigrin, glucoerucin and indolic 4-hydroxyglucobrassicin and lower co-efficients (and also lower contents) of glass-house feeding scores (at 7 and 13 days), neoglucobrassicin and gluconapin (**Table 1**). PC2 clearly separated YR Gold, BN4059, and BN4303 from Rubra and BN3383 as evident by their differential location in PCA-biplot (**Figure 7**) and by their contrasting mean PC scores (**Table 1**).

## Discussion

### Genotypic Differences in the Constitutive Glucosinolate Profiles

Glucosinolates (GSL) were quantified in the middle-aged leaves of eight different cabbage genotypes as DBM larvae mainly feeds on leaves; and younger leaves contain higher amount of glucosinolates compared to older leaves (Lambdon et al., 2003; Lambdon and Hassall, 2005). The amount of individual aliphatic-, indolic-, and total- glucosinolate contents varied constitutively across plant genotypes (**Figure 3**). Among the known 120 glucosinolate compounds in various plant species (Kliebenstein et al., 2001), only 11 were detected. No single genotype contained all these 11 glucosinolates and their contents ranged from traces to too high amounts. Besides the previous report of two aliphatic compounds (sinigrin and glucoiberin) and one indolic/ compound (glucobrassicin) dominating the glucosinolate patterns (Velasco et al., 2007; Cartea et al., 2008), this study found 4-hydroxyglucobrassicin at higher concentrations in few of the studied genotypes (**Figure 3**). This diversity in glucosinolate profiles in these eight genotypes is reasonable as the genotypes were developed from different parents (as per Asia Seed Company that provided these materials) and varies in terms of morphological features including variation in leaf size, shape, pigmentation and wax deposition etc. (**Figure 1**). The diversity of genotypes in glucosinolate contents and profiles rendered ideal materials for studying the preferential feeding of DBM.

### Diamondback Moths Preferred Certain Cabbage Lines

Insect herbivory is generally studied in common garden experiments that harness the natural field conditions to explore the feeding behaviors of various generalist and specialist insect species along with their natural enemies. Herbivory by a particular insect species is largely influenced by factors not limited to genotype (Bidart-Bouzat and Kliebenstein, 2008; Robin et al., 2016); leaf age and strata (Moreira et al., 2016) and host range (Zhang et al., 2012; Henniges-Janssen et al., 2014) along with potential influence of surrounding vegetation and insect population, biotic and abiotic stresses and environmental factors etc. (Hopkins et al., 2009; Grzywacz et al., 2010; Augusto and Amilton, 2014). We were particularly focused on interrogating the genotype specific feeding attributes of DBM larvae with regards to glucosinolate profiles and hence, opted for controlled experiments in both glass-house and *in vitro* conditions in a way that the non-genotypic factors can be kept to a minimum. Our tests identified BN4303, BN4059, and BN4072 as the least damaged (resistant) and BN3383, YR Gold and Rubra as most damaged (susceptible) genotypes (**Figures 4, 5**) in both field and laboratory tests. This results, however, should be viewed keeping the previous feeding experience of the insects in mind as the larvae were previously reared on *B. napus* leaves which may have an influence on the ability of insects to deal with plant toxins and their feeding preferences.

### Preferences Is not Related to Total Amount of GSL, but to GSL Profile

Glucosinolates, characteristic to *Brassica* and its crucifer relatives, are known to play differential role of both defense molecule (Mithen et al., 1995; Benderoth et al., 2006) and feeding and oviposition stimulants to a variety of insect pests and their predators (Li et al., 2000; Velasco et al., 2007; Müller et al., 2010). When the variable feeding preferences of DBM larvae were interrogated with the diverse glucosinolate profiles in eight cabbage genotypes, it became obvious that there is no linear association between the total glucosinolate contents and plant’s resistance against DBM. For example, despite having the least (4.9 μmol g^-1^ DW) and the most (157.6 μmol g^-1^ DW) total glucosinolate contents, respectively, the genotypes Ohgane and YR Gold didn’t show any obvious variation in insects feeding preference as both of the genotypes were severely damaged. On the other hand, the most resistant genotype BN4303 have comparatively less total glucosinolate (15.1 μmol g^-1^ DW). This adds to the reports of contrasting roles played by total glucosinolates in insect resistance (Hopkins et al., 2009; Sarosh et al., 2010; Santolamazza-Carbone et al., 2014). Higher contents of total glucosinolates were reported to be positively associated with higher DBM damage in *A. thaliana* (Bidart-Bouzat and Kliebenstein, 2008), increased visit from cabbage butterflies (*Pieris rapae*) in oilseed rape (Giamoustaris and Mithen, 1995) and negatively associated with specialists flea beetles (*Psylliodes convexior* and *Phyllotreta zimmermani*) damage in *A. thaliana* (Mauricio, 1998). Lower contents of total glucosinolates were reported to be associated with higher and lower pupal mass of *Pieris rapae* and adult body mass of *Pteromalus puparum*, respectively, in wild cultivars and cultivated varieties of *B. oleracea* (Hopkins et al., 2009). In most of the cases, a generalized notion is thus developed regarding the influence of total glucosinolate, either positively or negatively, on various generalists, specialists and their natural enemies (Lankau, 2007; Arany et al., 2008; Kos et al., 2011). Contrary to this, a few cases reported no direct association between high total glucosinolates and DBM damage in *B. juncea* and *A. thaliana* (Bodnaryk, 1997; Li et al., 2000; Arany et al., 2008).

Additionally, despite a few previous reports of contrasting roles of two classes of glucosinolates namely, aliphatics and indolics in conferring resistance or susceptibility against insect herbivory (De Vos et al., 2008; Gols et al., 2008; Newton et al., 2009; Kos et al., 2011; Bohinc et al., 2013), our PCA-based approach didn’t recognize any definitive patterns in the contents of either of these two classes of glucosinolates and feeding preference of DBM larvae. This observation suggested that DBM’s feeding preference is not restricted to any particular classes of glucosinolates in our study. Together, all these results of laboratory and field experiments indicated that the insect–herbivore interaction is dynamically dependent on the genotype specific glucosinolate profiles as evident by its differential influence on the feeding behavior and damage levels of various generalist and specialist insects (Mauricio, 1998; Li et al., 2000; Barth and Jander, 2006; Bohinc et al., 2013).

### Contrasting Glucosinolate Profiles in Resistant and Susceptible Genotypes

In an attempt to identify any underlying pattern hidden among the overall variability of feeding behaviors and glucosinolate contents in the studied eight genotypes we used PCA and observed a conspicuous contrasting profiles of certain glucosinolates between resistant and susceptible genotypes (**Table 1** and **Figure 7**). Glucobrassicin, glucoiberin, and glucoiberverin were only detected in resistant genotypes BN4303, BN4059, and BN4072 whereas in susceptible genotypes BN3383, Rubra, and YR Gold these glucosinolates were completely absent indicating their potential role in conferring resistance against DBM (Supplementary Figure S2). Conversely, 4-hydroxyglucobrassicin, glucoerucin, glucoraphanin, progoitrin, and gluconapin were absent in these three resistant genotypes whereas those were present at relatively high amount in susceptible genotypes (Supplementary Figure S2). These results indicated the role of glucobrassicin, glucoiberin and glucoiberverin as potential repellents and 4-hydroxyglucobrassicin, glucoerucin, glucoraphanin, progoitrin and gluconapin as potential attractants for DBM. Based on these results, an apparent conclusion can be made that both the presence of these potential repellents and absence of these potential attractants is needed simultaneously to achieve non-preference and hence, resistance against DBM in these genotypes (and vice-versa, for susceptibility). However, it is not clear if the presence of these potential repellents alone, irrespective of the presence of the potential attractants, can make these genotypes resistant as no genotypes were found to contain the glucosinolates of both these groups. Further research is thus needed to validate this phenomenon.

Among the three potential repellents identified in this study, indolic glucobrassicin and aliphatic glucoiberin was negatively associated with insect richness, herbivory and larval fitness (Gols et al., 2008; Poelman et al., 2009; Kos et al., 2011; Santolamazza-Carbone et al., 2014, 2016). Reports on the role of the other glucosinolate, glucoiberverin in against insect herbivory is, however, scarce. Our approach identified glucoiberverin to be negatively associated with the feeding preference but the concentration of this glucosinolate is trivial compared to the amounts of other glucosinolates in each of the resistant genotypes. Nevertheless, glucoiberverin was not present in the susceptible genotypes at all. This indicates toward the possibility that glucoiberverin, even in trace amounts is needed for resistance in these genotypes.

Sinigrin was detected at a relatively high amount (in fact, it led the glucosinolate profiles) in all of our less preferred genotypes. However, unlike the contrasting groups of potential repellents and attractants, it was also present in susceptible genotypes such as Rubra and YR Gold, in comparatively lesser amounts though. This further adds to the diverse reports of sinigrin which was associated with reduced herbivory (Bodnaryk, 1997; Brian Traw and Dawson, 2002; Agrawal and Kurashige, 2003; Santolamazza-Carbone et al., 2014) in one hand and increased body weight and infestation of *Mamestra brassicae* larvae on young plants of *B. oleracea* var. *acephala* (Santolamazza-Carbone et al., 2016); increased visit from *P. xylostella* on *B. juncea* under laboratory conditions (Spencer et al., 1999) on the other hand, making it an important glucosinolate in insect–herbivore interaction.

All these further add to the dynamic roles of glucosinolates in plant–herbivore interactions (Tierens et al., 2001; Mewis et al., 2005; Hopkins et al., 2009; Santolamazza-Carbone et al., 2014) and points toward the fact that insect resistance should be viewed holistically taking other associated factors such as other secondary metabolites (Van Loon et al., 2002); further downstream breakdown products (Reed et al., 1989; Roessingh et al., 1997; Van Loon et al., 2002; Bezemer and Vandam, 2005; Renwick et al., 2006); plant physiological attributes (Sarosh et al., 2010); physical and biological context of both plant and insect population (Bidart-Bouzat and Kliebenstein, 2008) into account.

These findings can be extended to identify the best glucosinolate profiles for the prevalent insect species and their natural enemies of a particular area. This will be helpful in identifying the common glucosinolate determinants of resistance and in selective breeding of cultivars with specific glucosinolate profiles that may provide resistance against the prevalent insect species and their natural enemies of that particular area.

## Conclusion

The PCA based approach of interrogating the feeding attributes with glucosinolate contents identified novel contrasting profiles of two sets of glucosinolates in the least and the most preferred genotypes of cabbage against DBM larvae that may act as potential repellents and attractants. These findings will be helpful for setting breeding priorities in developing cabbage varieties with specific glucosinolate profile that can provide resistance against DBM for areas where this pest developed resistance against pesticides. Additionally, the effects of these potential repellents and attractants on other insects of cabbage and their predators in natural field conditions can further be investigated. This will widen our understanding of the dynamic roles of glucosinolates in plant–insect interaction.

## Author Contributions

I-SN, J-IP, and AR conceived and designed the study. AR managed the experimental plants, reared insects, conducted feeding tests, and prepared samples for HPLC. MH and AR conducted statistical analysis and wrote the manuscript. HK conducted the HPLC analysis.

## Conflict of Interest Statement

The authors declare that the research was conducted in the absence of any commercial or financial relationships that could be construed as a potential conflict of interest.
