# Supplementary material for: Glucosinolate Profiles in Cabbage Genotypes Influence the Preferential Feeding of Diamondback Moth (Plutella xylostella)
Source: Front Plant Sci. 2017 Jul 18;8:1244. doi: 10.3389/fpls.2017.01244 (PMC5513964; doi:10.3389/fpls.2017.01244)
Supplement: Supplementary file 1 [file Data_Sheet_1.docx]

## Supplementary Figures and Tables


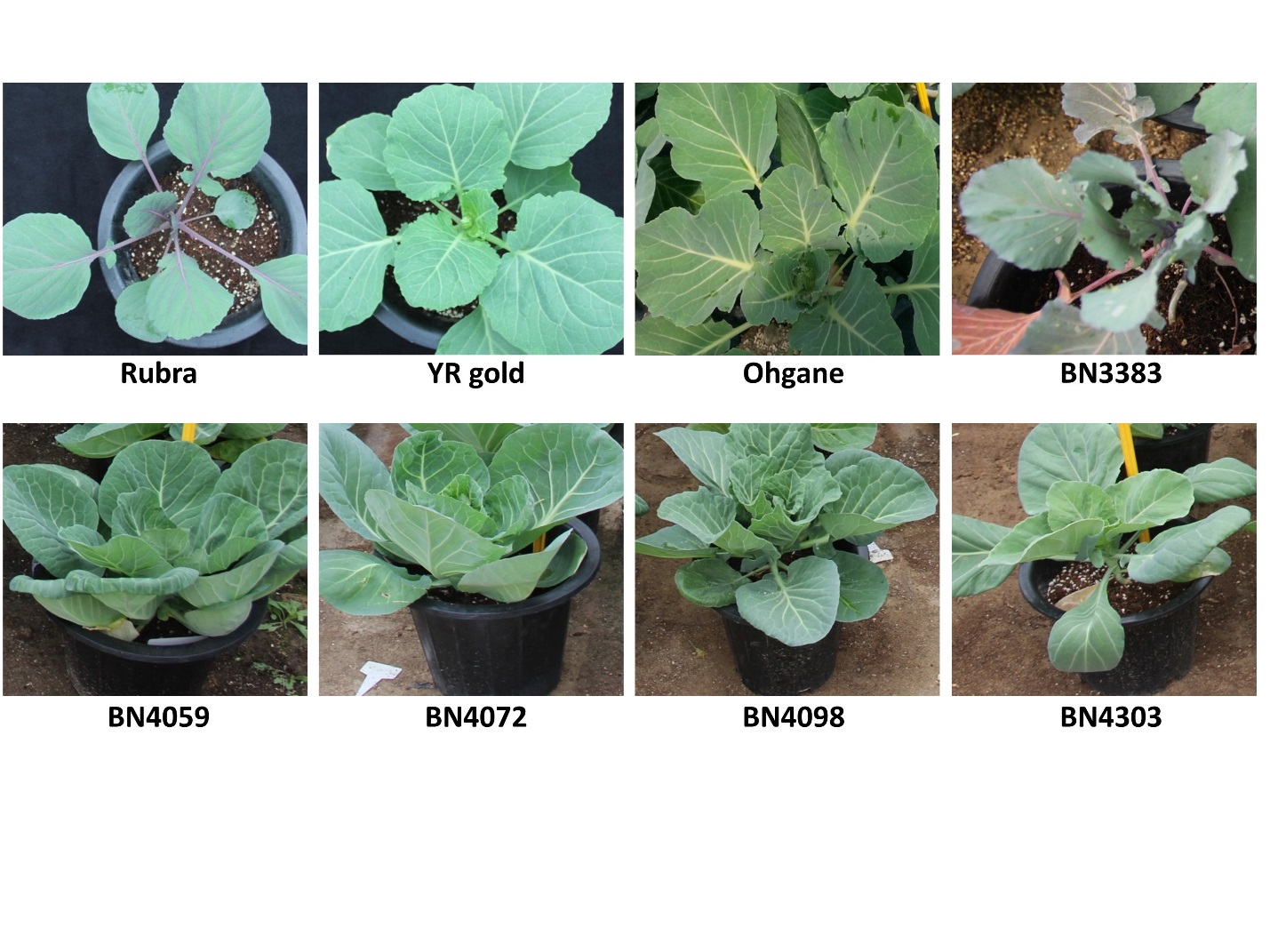


### **Figure S1.** Morphological appearance of eight cabbage genotypes with photos at the onset of Diamondback moth infestation. Rubra, YR gold and Ohgane are cabbage cultivars. The other three genotypes with ‘BN’ prefixes are the cabbage inbred lines.


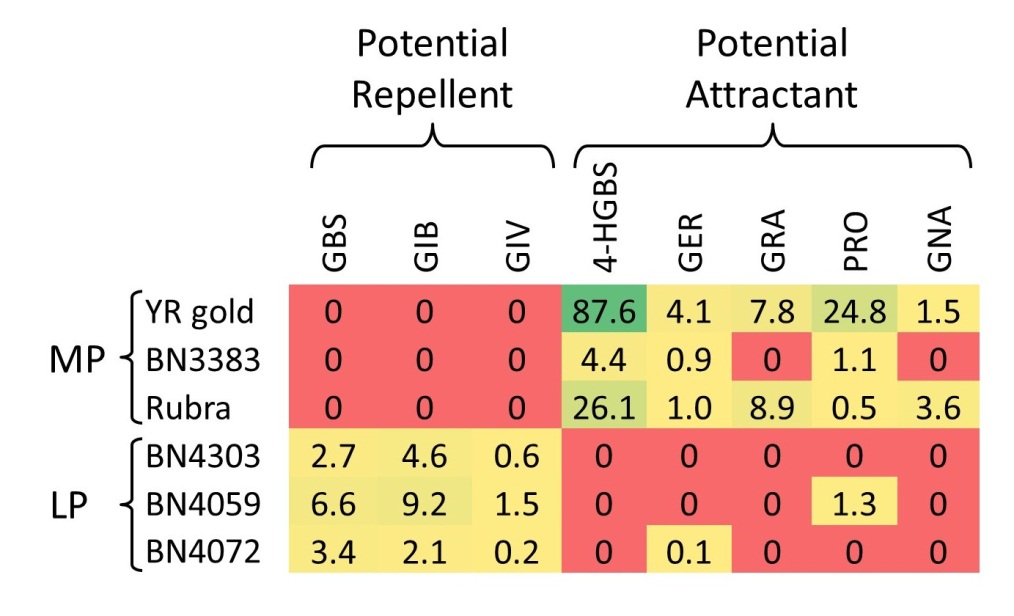


### **Figure S2.** Heatmap showing the contrasting contents of groups of glucosinolate compounds in most preferred (MP) and least preferred (LP) genotypes. Numerical values in heatmap represent glucosinolate contents in µmol g^-1^ dry weight.

### **Table S1.** Statistical procedure followed for analyzing data obtained from different measurement techniques.

| **Data type** | **Data source** | **Source of variation explored** | **Date presented in** | **Statistical analysis conducted** | **Post-hoc analysis**  **conducted** | **Software packages used** |
| --- | --- | --- | --- | --- | --- | --- |
| Glucosinolate content | HPLC measurements | Between genotypes | Figure 3 | One-way analysis of variance (ANOVA) | Tukey’s Pairwise Comparisons | Minitab v. 17 |
| Glass-house feeding scores | Visual scoring of damage caused by DBM | Between genotypes | Figure 4 | Non-parametric Mood’s Median test | Bonferroni’s pairwise comparisons | Minitab v. 17 |
| Number of larvae per leaf disc | Counting | Between genotypes, time-points within genotype and time-points across genotypes | Figure 5 & 6 | generalized linear model with Poisson distribution | Tukey’s pairwise comparisons | SPSS v. 24 |
| Percent leaf area damage | Visual estimation of damaged leaves | Between genotypes | Figure 5 & 6 | Two-way ANOVA | Tukey’s pairwise comparisons | Minitab v. 17 |

### Table S2. Normality and deviance of data, degrees of freedom, test statistic and p value of the different traits

| **Trait** | **Variable** | **Normality of data** | **Degrees of freedom** | **F value** | **P value** |
| --- | --- | --- | --- | --- | --- |
| Glucosinolate content | Glucoiberin | Normal | 7 | 342.74 | <0.01 |
|  | Progoitrin | Normal | 7 | 182.71 | <0.01 |
|  | Glucoraphanin | Normal | 7 | 32.79 | <0.01 |
|  | Sinigrin | Normal | 7 | 149.93 | <0.01 |
|  | Gluconapin | Normal | 7 | 77.47 | <0.01 |
|  | glucoiberverin | Normal | 7 | 113.81 | <0.01 |
|  | Glucoerucin | Normal | 7 | 135.20 | <0.01 |
|  | 4-hydroxyglucobrassicin | Normal | 7 | 124.61 | <0.01 |
|  | Glucobrassicin | Normal | 7 | 495.45 | <0.01 |
|  | 4-methoxyglucobrassicin | Normal | 7 | 7.08 | <0.01 |
|  | Neoglucobrassicin | Normal | 7 | 5.41 | <0.01 |
|  | Total glucosinolate | Normal | 7 | 192.84 | <0.01 |
| Percent damage | 6 h in Multiple-choice feeding test | Normal | 7 | 50.61 | <0.01 |
|  | 6 h in Two-choice feeding test | Normal | 7 | 59.63 | <0.01 |
|  | 12 h in Multiple-choice feeding test | Normal | 7 | 32.34 | <0.01 |
|  | 12 h in Two-choice feeding test | Normal | 7 | 57.35 | <0.01 |
| **Poisson test for Number of larvae** | | **Deviance** | **Degrees of freedom** | **Chi-square** | **P value** |
| Multiple-choice feeding test | Genotype | 13.2% | 1 | 27.74 | <0.01 |
|  | Time-point |  | 1 | 0.00 | 1.00 |
| Two-choice feeding test | Genotype | 2.3% | 1 | 4.41 | 0.036 |
|  | Time-point |  | 1 | 0.03 | 0.873 |
| **Mood’s Median Test for Glass-house feeding score** | | **Normality** | **Degrees of freedom** | **Chi-square** | **P value** |
| Glass-house feeding score | Day 2 | Normal | 7 | 44.05 | <0.01 |
|  | Day 4 | Normal | 7 | 37.35 | <0.01 |
|  | Day 7 | Normal | 7 | 37.86 | <0.01 |
|  | Day 13 | Normal | 7 | 39.32 | <0.01 |

### Table S3. The range values of glass-house feeding test scores. Data were recorded based on a visual score of 1 – 9 scale with ‘1’ and ‘9’ being scored for the least and for the least and highest area damaged by Diamondback moth in eight genotypes of cabbage respectively, at different days (d) after introduction of DBM larvae in cabbage plants. The higher scores indicate the higher preference and higher damage by DBM larvae on the cabbage lines. Median data analyzed using Non-parametric Mood’s Median test and presented as median scores of eight observations in Figure 4.

| **Gentoypes** | **2 d** | **4 d** | **7 d** | **13 d** |
| --- | --- | --- | --- | --- |
|  | **Range** | **Range** | **Range** | **Range** |
| Rubra | 3.0-5.0 | 3.0-7.0 | 7.0-9.0 | 9.0-9.0 |
| YR gold | 1.0-3.0 | 3.0-5.0 | 7.0-9.0 | 7.0-9.0 |
| Ohgane | 3.0-5.0 | 5.0-7.0 | 6.0-9.0 | 7.0-9.0 |
| BN3383 | 1.0-3.0 | 1.0-5.0 | 5.0-7.0 | 7.0-9.0 |
| BN4059 | 1.0-1.0 | 1.0-5.0 | 3.0-5.0 | 3.0-7.0 |
| BN4072 | 1.0-1.0 | 3.0-5.0 | 3.0-7.0 | 5.0-7.0 |
| BN4098 | 1.0-3.0 | 3.0-5.0 | 5.0-7.0 | 5.0-9.0 |
| BN4303 | 1.0-1.0 | 1.0-3.0 | 1.0-3.0 | 1.0-5.0 |

### Table S4. Physical properties of glucosinolates after mass spectrometry analysis (HPLC/MS, Agilent 1200 series, Agilent Technologies) identified in the leaf samples of *B. oleracea* subspecies by HPLC (Yi et al. 2016).

| **No.** | **Retention Time (min)** | **Names** | **Molecular Weight** | **Structure** |
| --- | --- | --- | --- | --- |
| 1 | 8.960 | Glucoiberin | 344 | [M + H]^+^ |
| 2 | 9.365 | Progoitrin | 310 | [M + H]^+^ |
| 3 | 9.633 | Glucoraphanin | 358 | [M + H]^+^ |
| 4 | 10.068 | Sinigrin | 302 | [M + Na]^+^ |
| 5 | 12.430 | Gluconapin | 316 | [M + Na]^+^ |
| 6 | 13.385 | Glucoiberverin | 350 | [M + Na]^+^ |
| 7 | 13.586 | 4-hydroxyglucobrassicin | 385 | [M + H]^+^ |
| 8 | 15.194 | Glucoerucin | 364 | [M + Na]^+^ |
| 9 | 16.250 | Glucobrassicin | 369 | [M + H]^+^ |
| 10 | 17.138 | 4-methoxyglucobrassicin | 399 | [M + H]^+^ |
| 11 | 19.399 | Neoglucobrassicin | 399 | [M + H]^+^ |
